# Regulation of V-ATPase Activity and Organelle pH by Phosphatidylinositol Phosphate Lipids

**DOI:** 10.3389/fcell.2020.00510

**Published:** 2020-06-23

**Authors:** Subhrajit Banerjee, Patricia M. Kane

**Affiliations:** ^1^Department of Molecular and Cell Biology, University of California, Berkeley, Berkeley, CA, United States; ^2^Department of Biochemistry and Molecular Biology, SUNY Upstate Medical University, Syracuse, NY, United States

**Keywords:** phosphatidylinositol phosphate, acidification, organelle, V-ATPase, PIKfyve, lysosome, Golgi apparatus, endosome

## Abstract

Luminal pH and the distinctive distribution of phosphatidylinositol phosphate (PIP) lipids are central identifying features of organelles in all eukaryotic cells that are also critical for organelle function. V-ATPases are conserved proton pumps that populate and acidify multiple organelles of the secretory and the endocytic pathway. Complete loss of V-ATPase activity causes embryonic lethality in higher animals and conditional lethality in yeast, while partial loss of V-ATPase function is associated with multiple disease states. On the other hand, many cancer cells increase their virulence by upregulating V-ATPase expression and activity. The pH of individual organelles is tightly controlled and essential for function, but the mechanisms for compartment-specific pH regulation are not completely understood. There is substantial evidence indicating that the PIP content of membranes influences organelle pH. We present recent evidence that PIPs interact directly with subunit isoforms of the V-ATPase to dictate localization of V-ATPase subpopulations and participate in their regulation. In yeast cells, which have only one set of organelle-specific V-ATPase subunit isoforms, the Golgi-enriched lipid PI(4)P binds to the cytosolic domain of the Golgi-enriched a-subunit isoform Stv1, and loss of PI(4)P binding results in mislocalization of Stv1-containing V-ATPases from the Golgi to the vacuole/lysosome. In contrast, levels of the vacuole/lysosome-enriched signaling lipid PI(3,5)P_2_ affect assembly and activity of V-ATPases containing the Vph1 a-subunit isoform. Mutations in the Vph1 isoform that disrupt the lipid interaction increase sensitivity to stress. These studies have decoded “zip codes” for PIP lipids in the cytosolic N-terminal domain of the a-subunit isoforms of the yeast V-ATPase, and similar interactions between PIP lipids and the V-ATPase subunit isoforms are emerging in higher eukaryotes. In addition to direct effects on the V-ATPase, PIP lipids are also likely to affect organelle pH indirectly, through interactions with other membrane transporters. We discuss direct and indirect effects of PIP lipids on organelle pH, and the functional consequences of the interplay between PIP lipid content and organelle pH.

## Phosphoinositides Contribute to Organelle Identity and Function

Unique enrichment of phosphoinositide phosphate (PIP) lipids across sub-cellular organelles helps to define their identity (Strahl and Thorner, 2007; Hammond et al., 2012). A general theme in the field of organelle biology is that the Golgi network is defined by the presence of PI(4)P, endosomes by PI(3)P, plasma membrane by PI(4,5)P_2_ and late endosome/lysosome by PI(3,5)P_2_ (Strahl and Thorner, 2007). There are exceptions to this generalization: the plasma membrane has a substantial amount of PI(4)P and late-endosome/lysosome also bears PI(3)P (Wallroth and Haucke, 2018). In addition, PI(4)P has been detected in endosomes and lysosomes (Hammond et al., 2014). The organelle network is more complicated in mammals, but maintains the same basic features (Idevall-Hagren and De Camilli, 2015). This review focuses primarily on PI(4)P, PI(3)P, and PI(3,5)P2 because of their role in V-ATPase function.

### Phosphatidylinositol 4-Phosphate, PI(4)P

Phosphorylation of phosphatidylinositol (PI) and interconversions of PIP lipids are driven by kinases, phosphatases, and lipid transfer proteins. PI(4)P is synthesized by site-specific kinases at the Golgi and plasma membrane (Balla et al., 2002, 2005). In yeast, Stt4 and Pik1 synthesizePI(4)P at the plasma membrane and the Golgi network respectively, the two PI(4)P-enriched locations in cells (Audhya et al., 2000); the mammalian orthologs PI4KIIIα and PI4KIIIβ play similar roles (Dornan et al., 2016). The lipid phosphatase Sac1 regulates pools of PI(4)P at the Golgi (Faulhammer et al., 2007; De Matteis et al., 2013). Vps74/GOLPH3 interactions with Sac1 help to determine the gradient of PI(4)P in the Golgi, with highest levels in the trans Golgi (Wood et al., 2012). ER-localized Sac1 at ER-plasma membrane contact sites acts in trans to regulate plasma membrane PI(4)P, via the activity of members of lipid transfer proteins in the oxysterol binding protein family, Osh and ORP proteins (Stefan et al., 2011; Dickson et al., 2016). PI(4)P plays multiple critical roles in the cell, regulating trafficking, driving distribution of other lipids, and acting as a precursor for the important signaling lipids PI(4,5)P_2_ and PI(3,4,5)P_2_ (Tan and Brill, 2014). In yeast, PI(4)P-deficient *pik1* mutants exhibit dysregulated trafficking from the trans-Golgi network, disorganization of actin, and impairment of the secretory pathway (Hama et al., 1999; Walch-Solimena and Novick, 1999). In addition, *pik1* mutants manifest a kinetic delay in maturation of pro-peptides destined to the vacuole from the Golgi (Audhya et al., 2000). In general, defects in sorting as a result of lower PI(4)P can be attributed to failure to recruit critical trafficking effectors to the membrane, such as AP-1and GGA proteins (Wang et al., 2003, 2007). The precise level of PI(4)P is also important, as elevation of PI(4)P in the Golgi of mammalian cells, as a result of RNAi of *SAC1*, causes mislocalization of Golgi glycosylation enzymes mannosidase-II and N-acetylglucosamine transferase-I (Cheong et al., 2010). Mutations causing elevation of Golgi PI(4)P also cause mislocalization of a Golgi membrane protein in yeast (Wood et al., 2012). PI(4)P has also emerged as an important determinant of distribution of other lipids and overall membrane structure, as OSBP family members use PI(4)P to power exchange for phosphatidylserine, cholesterol, and other lipids at membrane contact sites (Chung et al., 2015; Mesmin et al., 2017; Antonny et al., 2018; Nishimura et al., 2019). Some of the many defects arising from PI(4)P deficiency may arise from altered distribution of other lipids. Finally, plasma membrane PI(4)P acts as a precursor for PI(4,5)P_2_, a major organizer of plasma membranes, as well as the downstream signaling lipid PI(3,4,5)PP_3_ (Balla, 2013).

### Phosphatidylinositol 3-Phosphate, PI(3)P and Phosphatidylinositol 3,5-Bisphosphate, PI(3,5)P_2_

Phosphatidylinositol 3-Phosphate has a critical role in vesicular trafficking and protein sorting (Strahl and Thorner, 2007). PI(3)P synthesis at the endosome relies on the PI 3-kinase Vps34. PI(3)P synthesis is critical to sorting of vacuolar proteins (Schu et al., 1993; Stack et al., 1995), and also important for endosomal maturation and subsequent synthesis of PI(3,5)P_2_ in the late endosome and lysosome (Brown et al., 1995; Gary et al., 1998). PI(3)P is essential for autophagosome formation and maturation, and therefore regulates autophagy (Vergne and Deretic, 2010; Marat and Haucke, 2016). Vps34 participates in two different complexes that target these distinct cellular functions (Kihara et al., 2001). Protein sorting and membrane trafficking are mediated by an endosomal Vps34 complex that also contains Vps15, Vps30/Beclin and Vps38/UVRAG (Rostislavleva et al., 2015). Starvation induced autophagy (Kametaka et al., 1998) and cytosol to vacuole transport (Kim and Klionsky, 2000) require a complex of Vps34, Vps15, Vps30/Beclin, Vps38, Atg14, and Atg38/NRBF2 (Araki et al., 2013). PI(3,5)P_2_ is generated from PI(3)P and required for endo/lysosomal maturation, luminal acidification, and merging with multi-vesicular bodies (MVB) (Dove et al., 2002; Naufer et al., 2018; Wallroth and Haucke, 2018). PI(3,5)P_2_ controls organelle fusion and fission by regulating the Ca^2+^ concentration of organelle lumen (Dong et al., 2010). PI(3,5)P_2_ at the endo/lyososome is dynamically regulated by hyperosmotic stress, which offers protection from osmotic stress (Bonangelino et al., 2002; Jin et al., 2017). An intricate regulation of PI(3)P and PI(3,5)P_2_ might be responsible for endo/lysosomal positioning, trafficking and signaling (Wallroth and Haucke, 2018). PI(3,5)P_2_ appears to be regulated at the endo/lysosome in response to availability of glucose (Yordanov et al., 2019). This is accomplished by the nutrient-sensing kinase, AMPK, in collaboration with the BORC1 complex, a regulator of lysosome trafficking (Yordanov et al., 2019).

Endosomal sorting has recently been implicated in multiple diseases, illuminating the crucial role of the endosome as a sorting hub in humans (Ketel et al., 2016). In mammalian cells, PI(4,5)P_2_ is required for the early endocytic step and there is a PI(4,5)P_2_ to PI(3)P conversion during formation of early endosomes (Zoncu et al., 2009). From the endosomes, cargos are recycled to plasma membrane through recycling endosomes which require a switch of PI(3)P to PI(4)P (Ketel et al., 2016). In contrast, anterograde maturation of endosomes requires PI(3)P and eventually, PI(3,5)P_2_ in the membrane, as they become late endosomes and ultimately merge with lysosomes (Wallroth and Haucke, 2018). These dynamic PIP interconversions help to balance trafficking with maintenance of organelle integrity. Experimentally, acute conversion of PIPs using chemical-genetics, optogenetics, and single vesicle studies, have greatly advanced the understanding of this field (Zoncu et al., 2009; Idevall-Hagren et al., 2012).

### Interactions of PIPs With Cytosolic and Membrane Proteins

Much of the functional importance of PIPs derives from the recruitment of cytosolic proteins to specific membranes and the generation of PIP-specific conformational changes in integral membrane proteins. The majority of cytosolic or peripheral membrane proteins that bind PIPs bind through conserved PIP binding domains (Lemmon, 2008; Hammond and Balla, 2015). The pleckstrin homology (PH) domain is a well-characterized PIP binding domain that binds to PIP isoforms selectively (Lemmon, 2008). PH domains bind PIP head groups through deep pockets lined with basic amino acids, accounting for high-affinity (Naughton et al., 2018). Hydrophobic and aromatic amino acids lining the PIP binding site are thought to interact with the hydrophobic lipid bilayer (Lemmon, 2008). The FYVE domain (named after the PI(3)P binding proteins Fab1, YOTB, Vac1, and EEA1) (Burd and Emr, 1998; Lemmon, 2008) is another high-affinity lipid binding domain common to endosomal effectors. FYVE is a zinc finger domain bearing a binding site lined by basic amino acids that recognizes PI(3)P (Misra and Hurley, 1999). Other canonical PIP binding domains include PX, GRAM, and F-BAR domains (Lemmon, 2008). PROPPINS are a group of proteins represented by Atg18 and Hsv2 that contain a 7-bladed β-propeller that binds to PI(3)P and PI(3,5)P_2_ (Baskaran et al., 2012). The PIP binding site in PROPPINS does not conform to canonical PIP binding pockets. PROPPINS bind membranes containing PI(3)P or, PI(3,5)P_2_ via basic amino acid residues forming salt bridges with the PIP headgroup and aromatic amino acids that interact with the lipid bilayer (Baskaran et al., 2012). A group of cytosolic proteins known as MARCKS and MRP bind PI(4,5)P_2_ via long basic amino acid-rich patches (McLaughlin and Murray, 2005). These proteins bind predominantly by electrostatic interactions, and may present a less structured binding domain to the membrane.

Membrane proteins also bind PIPs, but in most cases, this binding does not involve canonical PIP-binding domains. The membrane proteins that are known to be regulated by PI(4,5)P_2_ include voltage-gated ion channels of the plasma membrane (Suh and Hille, 2005). These channels bind to PI(4,5)P_2_ using conserved cytosolic domains present in either their N- or C-terminal domains (Hilgemann, 2004; Suh and Hille, 2005). Mutagenesis, structural and domain swapping studies reveal that basic and hydrophobic residues are found in the PI(4,5)P_2_-binding domains in these channels (Hilgemann, 2004; Suh and Hille, 2005). The mammalian Ca^2+^/Na^+^ selective voltage and ligand-gated, two pore ion channels TPC1 and TPC2 are activated by PI(3,5)P_2_ (Boccaccio et al., 2014; Kirsch et al., 2018; She et al., 2018). A cryo-EM structure of TPC1 bound PI(3,5)P_2_ reveals that basic and polar amino acid residues from cytosolic loops and helices form the PIP binding site (She et al., 2018). Aromatic amino acids lying proximal and planar to the lipid bilayer also affect PI(3,5)P_2_ dependent regulation (She et al., 2018). In contrast, PI(3,5)P_2_ binding to the N-terminal domain of TRML1 channel (homologous to yeast Yvc1) requires multiple positively charged amino acid residues (Dong et al., 2010). Interestingly, TRPML1 channels localized transiently to the plasma membrane are inactive, suggesting that activity requires localization to PI(3,5)P_2_-containing compartments (Zhang et al., 2012).

## Luminal pH as an Identifying Feature of Organelles

### Organelles Have a Distinct Luminal pH

**FIGURE 1:**
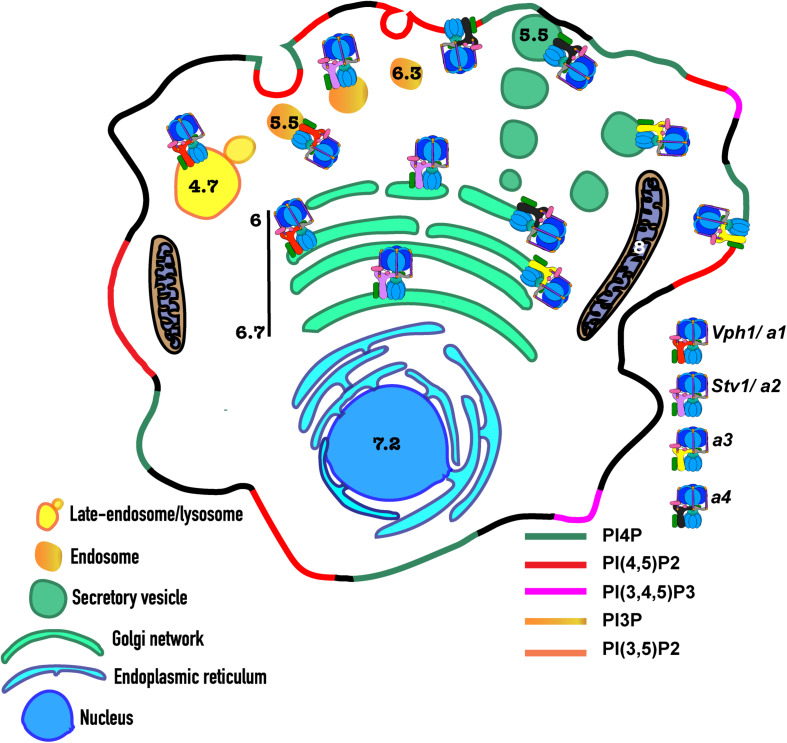
Distribution of V-ATPase isoforms, PIP lipids and the pH of subcellular organelles. Subcellular localization of isoforms of the 100 kDa a-subunit of the V-ATPase, in yeast and mammals, are indicated by separately coloring the a-subunit isoforms. The key to the respective a-subunit isoforms are indicated on the right side of the figure. On the left bottom, a key to the different organelles in the figure is present. The distinct enrichment of PIP lipids on the membranes of subcellular organelles and compartments are indicated by different colors. The key to the color-coded PIP species is on the bottom right side of the figure. Enrichment of PI(4)P is indicated in the compartments of the Golgi network and the plasma membrane. In addition to PI(4)P, the plasma membrane maintains an enrichment of PI(4,5)P_2_ and PI(3,4,5)P_3_. Secretory vesicles are also indicated to be enriched in PI(4)P. The endosomal compartments, including the early-, late- and recycling-endosomes and lysosomes, are characteristically enriched in PI(3)P. The late endosomes and lysosomes, in addition, maintains the highly regulated species PI(3,5)P_2_. pH or pH-ranges of all the different organelles are indicated by numbers inside or beside the respective organelles.

Organelles maintain a unique and tight range of pH, which also contributes to their identity (Figure 1; Casey et al., 2010). Measurement of organelle and cytosolic pH depends heavily on pH-dependent ratiometric fluorescent probes (Llopis et al., 1998; Martinez-Munoz and Kane, 2008; Casey et al., 2010; Diakov et al., 2013). The cytosol, nucleus and the endoplasmic reticulum (ER) maintain a near-neutral pH of approximately 7.2 (Grinstein et al., 1986; Paroutis et al., 2004; Casey et al., 2010; Tarsio et al., 2011). The Golgi network maintains a pH of roughly 6.7–6, gradually descending from the *cis-* to *medial*- to *trans-*Golgi compartments (Paroutis et al., 2004; Deschamps et al., 2020). Secretory granules are generally more acidic, with a pH range of 5.2–5.7 (Geisow and Evans, 1984; Casey et al., 2010). Although organelle pH ranges are generally conserved among eukaryotes, luminal pH can vary among tissues and organisms. The endocytic pathway is composed of early, recycling, and late endosomes and lysosomes. Endosomes vary in their pH, with the pH of early and recycling endosomes in the range of 6.3–6.5 (Casey et al., 2010; Mitsui et al., 2011), and late endosomes with a more acidic pH of 5.5 (Casey et al., 2010; Tarsio et al., 2011). MVB, responsible for targeting of some lysosomal and vacuolar proteins, targeting of autophagic cargo to the late-endosome/lysosome, and secretion of exosomes, are thought to maintain an acidic pH of ∼6 (Clague and Urbe, 2008), but the precise pH of MVB is yet to be determined. The lysosome is the most acidic organelle with a pH range of 4.7–5.5 in mammals (Casey et al., 2010; Tarsio et al., 2011). The pH of lysosomes can vary depending on their position with respect to the nucleus, with peripheral and central lysosomes possibly serving different functions (Johnson et al., 2016; Pu et al., 2016). Yeast vacuoles have many similarities to lysosomes in higher organisms, but vacuoles maintain a higher pH range of 5.5–6.2 (Tarsio et al., 2011), and vacuolar pH can vary substantially in response to extra- and intra-cellular cues (Martinez-Munoz and Kane, 2008; Diakov and Kane, 2010; Brett et al., 2011).

### Luminal pH and Organelle Function

Luminal pH is closely tied to organelle function. The strongly acidic environment of lysosome and vacuoles is optimal for the activity of various hydrolytic enzymes such as proteases (Li and Kane, 2009; Mauvezin and Neufeld, 2015). The pH gradient across lysosomes and vacuoles is utilized to drive transport of ions and metabolites in and out of lumens of these hydrolytic and storage organelles (Li and Kane, 2009; Carmona-Gutierrez et al., 2016). Chemical agents and mutations that alkalinize the Golgi lumen dysregulate protein glycosylation, impair transport, and alter Golgi morphology (Axelsson et al., 2001; Maeda et al., 2008). Mutations in a Golgi subunit-isoform of the V-ATPase (ATP6V0A2) affect Golgi acidification and cause the rare congenital human disease cutis laxa (Kornak et al., 2008; Farsi et al., 2016). The exocytosis machinery, particularly regulated secretory granules, require adequate acidification to perform efficient secretion. Chromaffin granules maintain an acidic pH (pH ∼5.5), and it is proposed that luminal pH is sensed in order to discriminate vesicles loaded and ready for secretion (Poea-Guyon et al., 2013). Acidification of synaptic vesicles [with pH ranging from 6.4 to 7 (Farsi et al., 2016, 2018)] is key to neurotransmission. Loading of neurotransmitters into synaptic vesicles exploits both the pH gradient across their membrane and membrane potential established by the V-ATPase proton pump to load neurotransmitters (Farsi et al., 2016). Additionally, synaptic vesicle pH appears to control neurotransmitter release, subsequent to loading (Hiesinger et al., 2005; Poea-Guyon et al., 2013; Bodzeta et al., 2017).

Endosomes are key protein and membrane sorting compartments. Release of ligands from receptors is regulated by endosomal pH, with some receptors, such as transferrin receptor, readily recycled back to the plasma membrane, while other receptor-ligand complexes such as EGF receptor proceed to the lysosome for degradation (Futter et al., 1996; Authier and Chauvet, 1999). Some receptors transmit distinct signals from endosomes (Bergeron et al., 2016). Multiple viruses, including influenza and coronaviruses, exploit the acidic environment of endosomes to drive conformational changes required for infection (Lakadamyali et al., 2004; Cossart and Helenius, 2014). Phagosomes become progressively more acidic as they enter the cell, and this acidification is important to their function in innate immunity (Sun-Wada et al., 2009). pH homeostasis in the endolysosomal pathway is increasingly implicated in metabolic control, proteostasis, aging, neuro-protection, adaptive immunity and inflammation (Carmona-Gutierrez et al., 2016; Lassen et al., 2016; Bohnert and Kenyon, 2017).

Finally, the plasma membrane plays a critical role in cellular pH homeostasis. A network of transporters, exchangers, and ATPases is dedicated to maintenance of cytosolic pH in the face of changing extracellular environment (Grabe and Oster, 2001). Tight control of cytosolic pH is essential in mammalian cells, where cytosolic acidification is a trigger for apoptosis (Sergeeva et al., 2017). Rapidly growing cancer cells are often highly dependent on acid-producing glycolytic metabolism and remodel the plasma membrane proteome to support H^+^ export, but at the same time establish an acidic extracellular environment optimal for metastasis (Stransky et al., 2016). In contrast, in some contexts, including distal renal tubule, epididymis, inner ear and osteoclasts, proton export is essential to achieve physiological functions, such as urine acidification, spermatogenesis, hearing, and bone resorption, respectively (Borthwick and Karet, 2002; Breton and Brown, 2013).

## V-ATPase—Evidence for Direct Interaction With PIP Lipids

### V-ATPase Structure and Function

**FIGURE 2:**
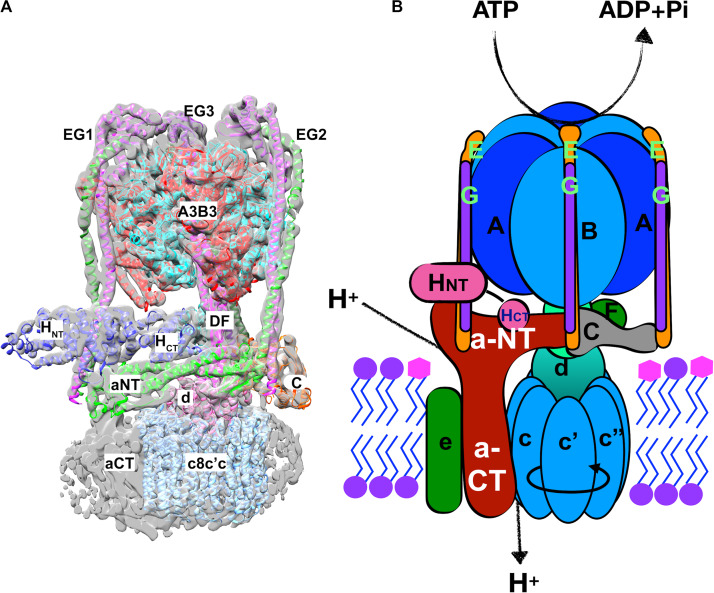
Structure of the yeast V-ATPase holoenzyme. **(A)** Cryo-electron micrograph structure of the assembled yeast V_1_-V_o_ complex (Zhao et al., 2015). A combination of the electron density and a chain trace of the different subunits is used to demonstrate the structural features of the V-ATPase holoenzyme. The subunits and distinct domains are indicated. The three distinct EG heterodimers (green and purple) are indicated by numbers on the side of the respective heterodimers. Upper-case letters indicate the subunit of the V_1_ sector and lower-cases represent the V_o_-sector subunits. **(B)** A cartoon of the V-ATPase holoenzyme together with the functions performed by the V_1_ and the V_o_ sectors is demonstrated. Precisely, the V_1_ sector performs the ATP hydrolysis which is coupled to the proton transport function of the V_o_ sector. The direction of rotation of the rotor subunits (c8c′c″dDF) is indicated by a circular arrow on the hetero-decameric c-ring (cc′c″).

V-ATPases are central players in organelle acidification and cellular pH control. V-ATPases are highly conserved, multisubunit proton pumps comprised of a peripheral membrane subcomplex called the V_1_ sector, and an integral membrane subcomplex called the V_o_ sector. The structure and subunit composition of the yeast V-ATPase is shown in Figure 2. Hydrolysis of cytosolic ATP occurs alternately at three catalytic sites in the V_1_A subunits, driving rotation of the central DF rotor stalk. The DF rotor stalk is attached to V_o_ d subunit and the ring of c-subunits in the V_o_ sector, and rotation of the c-ring relative to the integral membrane C-terminal domain of the a-subunit (aCT) promotes proton transport from the cytosol into the organelle lumen or to the cell exterior. Productive coupling of ATP hydrolysis and proton pumping relies on the AB hexamer in V_1_ and the V_o_ a-subunit remaining stationary as the central stalk rotates. Three stator stalks, each containing an EG heterodimer, connect the AB hexamer to V_o_, both through direct interactions with the N-terminal domain of the a-subunit (aNT) and via indirect interactions through the H and C subunits of V_1_.

**FIGURE 3:**
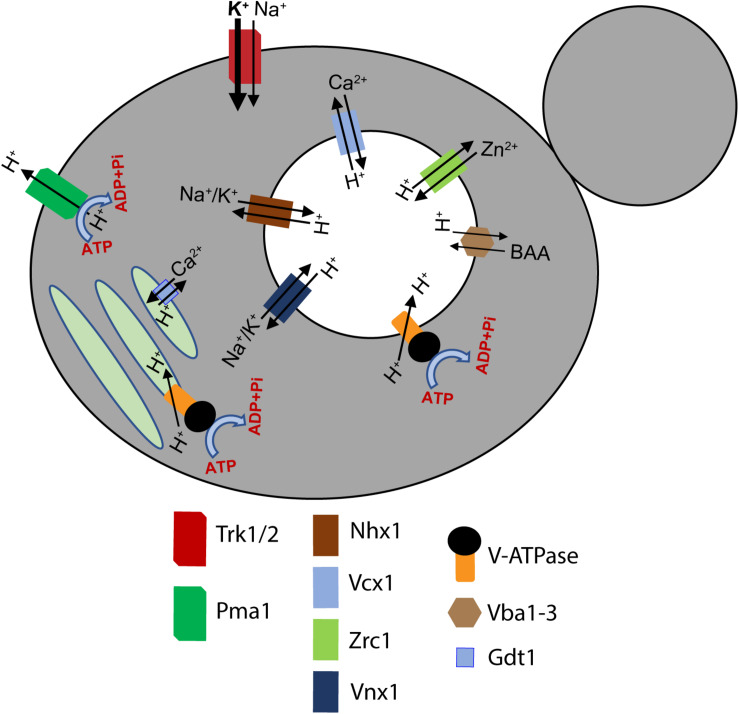
A unified model of proton pumps, transporters, and H^+^/ion exchangers that dynamically regulate the pH of subcellular compartments in yeast. A holistic regulation of pH of the cytosol, the vacuole, and the Golgi network is maintained by the coordinated transport of several ions. The plasma membrane Pma1 (green), vacuolar V-ATPase complex (chrome and black), and the Golgi V-ATPase complex (chrome and black) pump H^+^ out of the cytosol into the extracellular milieu, the vacuole, and the Golgi, respectively. Simultaneously, different ion transporters and H^+^/ion exchangers dynamically regulate the pH of the organelles and the cytosol (Li and Kane, 2009; Yenush, 2016). A key identifying the H^+^ pumps, ion channels, and H^+^/ion exchangers is provided at the bottom of the figure. BAA refers to basic amino acids. The arrowheads indicate the direction of ion transport.

V-ATPases are ubiquitous pumps found in multiple organelles of virtually all eukaryotic cells, but their activities in different locations are regulated at multiple levels. First, several subunits can be expressed as different isoforms that show tissue- and/or organelle-specific distribution (Marshansky and Futai, 2008). These isoforms can affect catalytic properties of the pumps they contain, determine their cellular localization, and support different regulatory mechanisms. Second, V-ATPases of multiple organisms have the capacity for reversible disassembly in response to diverse signals (Parra et al., 2014; Stransky and Forgac, 2015; Liu et al., 2017; McGuire and Forgac, 2018). In response to signals for disassembly of the V-ATPase, the V_1_ sector is detached from the V_o_ sector, accompanied by downregulation of both ATP hydrolysis and proton transport. Disassembly was first observed in yeast and in the tobacco hornworm Manduca sexta, and in both settings, occurred under conditions of glucose deprivation (Kane, 1995; Sumner et al., 1995). In V_1_ subcomplexes detached from V_o_, ATPase activity is inhibited (Parra et al., 2000) and V_o_ subcomplexes are closed to protons (Couoh-Cardel et al., 2015). Upon restoration of nutrients, V-ATPases were reassembled and activated. Since that time, it has become clear that reversible disassembly can occur in response to multiple signals, suggesting that the ability to reversibly disassemble is a general feature of eukaryotic V-ATPase structure (Trombetta et al., 2003; Li et al., 2014; Bodzeta et al., 2017; Liu et al., 2017; Oot et al., 2017; McGuire and Forgac, 2018). Isoform content can affect the ability of V-ATPases to disassemble (Kawasaki-Nishi et al., 2001b). Third, V-ATPases can be regulated by interactions with many regulatory proteins. These include glycolytic enzymes such as phosphofructokinase and aldolase (Lu et al., 2007; Su et al., 2008), which may couple V-ATPase activity to metabolic rate, regulators encoded by pathogens (SidK) (Zhao et al., 2017), and ARNO and Arf6, which help couple trafficking to endosomal pH sensing via the V-ATPase (Hurtado-Lorenzo et al., 2006). Finally, as electrogenic proton pumps, V-ATPases are highly sensitive to the activity of other transporters and their overall membrane environment. In many cellular contexts, V-ATPase activity can be limited by other electrogenic transporters creating an inside positive potential across organelle membranes, or can require transport of Cl^–^ or other anions to help balance the membrane potential. In addition, other transporters exploit the proton gradient created by V-ATPases to drive transport of other molecules (Figure 3). Collectively, the net proton export activity of these transporters is referred to as the “proton leak,” since it opposes the activity of the V-ATPase and results in a higher luminal pH than would be expected from the activity of the V-ATPase alone. Thus, both the activity of the V-ATPase itself and the final luminal pH are very sensitive to membrane environment.

The V_o_ a-subunit is central to V-ATPase regulation. V_o_ a-subunits show the highest level of isoform diversity in many organisms. For example, the V_o_ a-subunit is the only subunit present as isoforms in the yeast *Saccharomyces cerevisiae*, where the Stv1 a-subunit isoform resides predominantly at the Golgi apparatus and the Vph1 isoform is predominantly at the vacuole (Manolson et al., 1994). In humans, there are four a-subunit isoforms; one (a4) shows strict tissue specificity, while the others (a1, a2, and a3) are ubiquitously expressed but enriched in certain cell types. Organelle-specific enrichment is shown in Figure 1. The a-subunits are comprised of an N-terminal domain (aNT) exposed to the cytosol, and a C-terminal domain (aCT) that participates directly in proton transport (Mazhab-Jafari et al., 2016; Roh et al., 2018). Because yeast cells contain a single pair of isoforms, structural and functional differences of the two yeast a-subunit isoforms have been studied extensively. Chimeras of the aNT and aCT domains of the two yeast a-subunit isoforms revealed that information for targeting and reversible disassembly of V-ATPases appears to reside in the aNT domains, while the aCT domain dictates efficiency of coupling of ATP hydrolysis and proton transport (Kawasaki-Nishi et al., 2001a). More recently, structural studies of yeast V-ATPases containing either Vph1 or Stv1 revealed very little structural difference in overall backbone structures between the complexes at 6.6–6.7 A resolution (Vasanthakumar et al., 2019). Structures of the Vph1- and Stv1-containing V_o_ domains at higher resolution (3.1–3.2 A) have been obtained by cryo-EM (Roh et al., 2018; Vasanthakumar et al., 2019). Again, the backbone structures proved to be very similar, even though distinct catalytic properties are observed between purified V-ATPases containing the two isoforms. Despite the fact that Vph1 and Stv1 are only 49.4% identical in protein sequence, these data indicate that the a-subunit isoforms occupy very similar positions in the V-ATPase, and more subtle, side-chain differences may account for the differences they impart to their V-ATPase complexes.

### The aNT Domain as a Regulatory Hub

**FIGURE 4:**
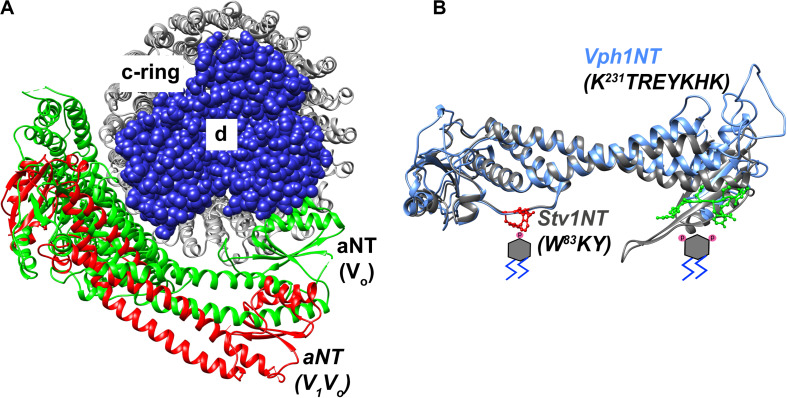
Structural features of the a-subunit. **(A)** A cryo-EM based model indicating the dynamic structural re-orientation of the N-terminal domain of the a-subunit in an assembled and active holoenzyme-conformation, colored in *red* (Zhao et al., 2015), and a disassembled and autoinhibited conformation in the isolated V_o_ sector, colored in *green* (Stam and Wilkens, 2017). The chain traces of the c-ring comprized of c8c′c″ is colored in gray and the spherical atoms of the d-subunit is colored in blue. **(B)** A superimposition of the cryo-EM structure of the NT-domain of Vph1 in *blue* (Zhao et al., 2015) and a Phyre2 based structural model of the Stv1-NT in *gray*. Stv1NT was modeled to the available low-density structure of the Stv1-V-ATPase (Vasanthakumar et al., 2019). PI(4)P and PI(3,5)P_2_ molecules are indicated with phosphates drawn in pink on the respective positions of a phosphatidylinositol lipid below the proximal and distal subdomains of the aNT domain, respectively. The PI(4)P binding site in Stv1, comprized of a W^83^KY sequence in the proximal end of the Stv1NT domain is indicated using *red* ball-and-stick atomic side chains. A PI(3,5)P_2_ recognition site in Vph1, comprized of a K^231^TREYKHK sequence in the distal end of the Vph1NT domain is indicated using *green* ball-and-stick atomic side chains.

Substantial evidence indicates that the aNT domain of a-subunit isoforms is a regulatory hub within the pump. A number of regulatory interactions with other proteins, such as the glycolytic enzyme aldolase, are proposed to involve this domain (Lu et al., 2004). In addition, as shown in Figure 2, the aNT domain falls at the interface of the V_1_ and V_o_ subcomplexes, and interacts with V_1_ subunits at two distinct stator stalks. The aNT domain itself is an extended structure, with two globular domains joined by a coiled-coil (Figure 4). The globular domains have been designated the “proximal domain,” which contains the N-terminus and the region adjacent to the first transmembrane domain, and the “distal domain” which is at the opposite end of the coiled coil (Oot et al., 2017). The available structures indicate significant differences in the position of the aNT domain in intact V-ATPase complexes vs. isolated V_o_ structures, and these differences impact both the proximal and distal domains (Figure 4). Specifically, the aNT collapses toward the V_o_ d-subunit of the central stalk in V_o_ structures and is pulled away from this stalk by interactions with the stator stalks in intact V-ATPase structures (Stam and Wilkens, 2016, 2017; Vasanthakumar et al., 2019). These structures suggest that adjusting the position of the aNT domain may be a critical step in reassembly of the V-ATPase after glucose deprivation or other triggers inducing reversible disassembly (Oot et al., 2017). It is also possible that the aNT assumes intermediate conformations that could result in partial destabilization of the V-ATPase (Li et al., 2014). Both the proximal and distal domains contain membrane-adjacent loops that are poorly conserved and often poorly resolved in the cryo-EM structures. The position of these loops suggest that they could be candidates for interaction with the cytosolic face of the membrane, but it is notable that they contain no established lipid binding motifs.

### Vacuolar V-ATPases and PI(3,5)P_2_

As described above, the low level signaling lipid PI(3,5)P_2_ is enriched in late endosomes and lysosomes/vacuoles. Yeast mutants defective in PI(3,5)P_2_ synthesis (*fab1* and *vac14* mutants) have greatly enlarged vacuoles that are defective in uptake of the quinacrine, a fluorescent weak base, suggesting a defect in acidification (Gary et al., 1998). Ratiometric fluorescence measurements indicated a vacuolar pH of 7 in *fab1* mutants vs. a pH of 6 in wild-type cells (Yamamoto et al., 1995). Subsequent experiments indicated that vacuolar pH could be maintained at wild-type in the absence of PI(3,5)P_2_ (Ho et al., 2015). V-ATPase activity and proton pumping were assessed in vacuolar vesicles isolated from *fab1* and *vac14* mutants (Li et al., 2014). Both activities were approximately 50% of that in wild-type; this may well be sufficient to maintain vacuolar pH in the absence of pH challenges. The levels of Vph1 were comparable in wild-type and mutant vacuoles, suggesting that there was not a defect in trafficking of V_o_ subunits to the vacuolar membrane. V_1_ subunits were present at wild-type levels in the mutants, but at reduced levels at the vacuolar membrane, suggesting a defect in biosynthetic assembly or stability of the V-ATPase complex. Interestingly, salt stress, which transiently increases PI(3,5)P_2_ by up to 20-fold, generated an almost twofold increase in V-ATPase activity, accompanied by increased levels of V_1_ subunits, in wild-type vacuolar vesicles (Li et al., 2014). This response was missing in vacuoles from salt-treated *vac14* mutant vacuoles. These data suggested that PI(3,5)P_2_ is not absolutely required for V-ATPase assembly or activity, but is required for full activity and for activation in response to osmotic challenge.

Preliminary evidence suggested that Vph1-containing V_o_ domains might bind to PIP lipids, so the N-terminal domain of Vph1 (Vph1NT) was visualized as a GFP fusion. The Vph1NT-GFP fusion was cytosolic in wild-type cells, but was recruited to membranes when PI(3,5)P_2_ levels were increased transiently by salt shock or constitutively in a *FAB1* mutant that maintains high levels of PI(3,5)P_2_ (Li et al., 2014). Significantly, replacing full-length Vph1 with the Vph1NT mutant abolishes V_o_ domain assembly and the V_1_ sector does not bind to Vph1NT (Jackson and Stevens, 1997; Diab et al., 2009). Therefore, these data suggested that Vph1NT has the capacity to interact with membranes in response to PI(3,5)P_2_ levels, and could bind to the lipid directly or indirectly. PI(3,5)P_2_ activation of the V-ATPase was pursued biochemically by assessing activation of V-ATPase activity in isolated vacuolar vesicles by short chain lipids (Banerjee et al., 2019). Short chain PI(3,5)P_2_ activated both ATPase and proton pumping activity of wild-type vacuolar vesicles, while short chain PI(4)P and PI(3)P provided little or no activation. Mutations in loops in both the proximal and distal domains of Vph1NT were designed that disrupt basic residues predicted to point toward the outer leaflet of the membrane and were not conserved in Stv1NT (Banerjee et al., 2019). These mutations were introduced into full-length Vph1. Two sets of mutations, both in the distal domain of Vph1NT (Figure 4), prevented activation by short chain PI(3,5)P_2_ in isolated vacuolar vesicles, while preserving wild-type basal V-ATPase activity (Banerjee et al., 2019). Cells containing these mutations exhibited no growth defect in the absence of stress, but had significant growth defects in a strain sensitized by deletion of the Hog1 stress-responsive transcription factor, as described below.

### Golgi V-ATPases and PI(4)P

As described above, the Golgi apparatus is enriched in PI(4)P, and in yeast, V-ATPases containing the Stv1 a-subunit isoform are localized to the Golgi at steady state. Remarkably, Stv1NT localizes constitutively to puncta, and this localization is lost when Golgi PI(4)P levels are reduced in a *pik1*^*ts*^ mutant, suggesting that Stv1NT binds to PI(4)P in vivo (Banerjee and Kane, 2017). *In vitro* experiments with bacterially expressed Stv1NT supported direct and specific binding to PI(4)P both on PIP blots and in liposome flotation assays. Potential PIP binding mutations were designed as described above, and a single point mutation (Figure 4) in the proximal domain of Stv1NT almost completely abolished flotation of Stv1NT with PI(4)P-containing liposomes *in vitro* (Banerjee and Kane, 2017). This mutation (K83A) had been previously characterized as affecting retrieval of Stv1-containing V-ATPases from the endosome to the Golgi (Finnigan et al., 2012). Consistent with those data, the mutant Stv1-containing V-ATPases escaped from the Golgi to the vacuole. A similar effect was seen for Stv1-V-ATPases in a *pik1*^*ts*^ mutant, suggesting that loss of PI(4)P binding affects Golgi retention of V-ATPases (Banerjee and Kane, 2017). Stv1-containing V-ATPases were recently purified for cryo-EM studies, and addition of PI(4)P resulted in a 1.9-fold increase in ATPase activity (Vasanthakumar et al., 2019). These data indicate that PI(4)P binding to Golgi V-ATPases is important for both their activity and localization in yeast.

These experiments suggest both structural correlates that can be applied to other V-ATPases and a potential range of physiological functions for PIP binding to V-ATPases. The strategy of predicting potential binding sites based on: (1) position of side chains relative to the membrane in structures or models, (2) regions of limited homology between isoforms, and (3) the presence of basic residues or clusters is consistent with PIP binding sites for other membrane proteins. The experiments with the yeast subunit isoforms suggest that PIP binding can affect activity of V-ATPases, and stabilize V_1_-V_o_ interactions (Li et al., 2014). Interestingly, the potential binding sites identified in Vph1NT and Stv1NT are present at opposite ends of the aNT domain. The mechanistic basis of V-ATPase activation by PIP lipids is not yet clear, but structural information about aNT positioning in isolated V_o_ sectors vs. intact V-ATPases suggests some possible mechanisms. As shown in Figure 4, cryo-EM structures indicate that both the proximal and distal domains of Vph1NT collapse from a more peripheral position dictated by interactions with the peripheral stator stalks in fully assembled V-ATPases toward a more central position, near the central stalk V_o_ d subunit, in isolated V_o_ subcomplexes (Figure 4). Interactions with PIP lipids could potentially help stabilize aNT domains in a more peripheral position similar to that seen in the fully assembled, active enzyme.

Although it is not possible to directly identify orthologs of Vph1 and Stv1 in mammalian V-ATPases, there is good evidence that the human a2 isoform is important in Golgi function, since loss of function in this subunit leads to phenotypes associated with loss of Golgi function (Kornak et al., 2008). Consistent with the results with yeast Stv1, the expressed human a2NT proved to bind specifically to PI(4)P-containing liposomes in a lipid flotation assay (Banerjee and Kane, 2017). Interestingly, a2NT does not contain the equivalent of the Stv1 K84 that was required for PI(4)P binding, suggesting that other areas of the protein support binding. However, this result suggests that a2-containing V-ATPases in the Golgi could respond to the presence of PI(4)P. As structures of higher eukaryotic V_o_ a subunit isoforms and improved models become available, it should be possible to address potential PIP binding sites and test their functional importance by methods similar to those used in yeast. These experiments are motivated by multiple connections between PIP lipids and pH regulation that may directly or indirectly involve direct interactions with the V-ATPase.

## Can PIP Lipids Affect V-ATPase Function *In Vivo*?

The effects of mutations that compromise PIP binding to V-ATPase subunit isoforms suggest that PIP binding can affect V-ATPase function in multiple ways. PI(4)P appears to help retain Stv1-containing V-ATPases in the Golgi, but also activates these V-ATPases *in vitro*. This suggests that PI(4)P availability may both activate Stv1-containing V-ATPases when they reach the Golgi, their primary organelle of residence, and help to maintain their localization there. In this context, PI(4)P binding to the V-ATPase Golgi-specific isoform plays a fundamental role in organelle identity. Effects of PI(3,5)P_2_ on Vph1-containing V-ATPases may be more complex, perhaps reflecting the function of PI(3,5)P_2_ as a low-level signaling lipid responsive to multiple signals. Assembled V_o_ domains are present at wild-type levels in mutants lacking PI(3,5)P_2_, suggesting that neither localization nor retention of the V-ATPase requires this lipid (Li et al., 2014). Vacuolar vesicles from mutants lacking PI(3,5)P_2_ have reduced activity and assembly, suggesting that PI(3,5)P_2_ may activate the V-ATPase by stabilizing binding of the V_1_ sector to V_o_ (Li et al., 2014). Furthermore, mutations in Vph1 that abolish activation by PI(3,5)P_2_ do not affect basal V-ATPase activity (Banerjee et al., 2019). These data suggest that PI(3,5)P_2_ interactions with Vph1 may be most important under conditions of stress. Under conditions of osmotic or salt stress, the vacuole serves as a first line of defense, protecting the cytosol from high levels of ions such as Na^+^ through the action of Na^+^/H^+^ antiporters before protective transcriptional responses, from pathways such as the Hog MAP kinase pathway, can be activated (Li et al., 2012). These antiporters operate at the expense of the vacuolar pH gradient (Li et al., 2012), creating a need for V-ATPase activation that may be fulfilled by PI(3,5)P_2_ synthesis and V-ATPase activation. Both PI(3,5)P_2_ synthesis and V-ATPase activity are activated by salt, and this response is entirely missing in *fab1Δ* and *vac14Δ* mutants (Bonangelino et al., 2002; Li et al., 2014). *vph1* mutants defective in PI(3,5)P_2_ activation have no obvious growth defects, but show synthetic phenotypes in combination with *hog1Δ* mutants (Banerjee et al., 2019). This suggests an important stress-dependent role for PI(3,5)P_2_ activation of V-ATPases that may operate in other contexts as well. It should be noted, however that although PI(3,5)P_2_ also promotes vacuolar acidification in plants, it does not act by increasing V-ATPase activity. Instead patch clamp experiments on isolated vacuoles indicate that PI(3,5)P_2_ acts as a negative regulator of the anion/H^+^ exchanger CLC-a (Carpaneto et al., 2017) thus limiting H^+^ export from the vacuole.

One of the prominent effects of loss of PI(3,5)P_2_ synthesis is formation of a greatly enlarged vacuole in yeast and cytosolic vacuolation in mammalian cells (Gary et al., 1998; Chow et al., 2007; Zhang et al., 2007). This phenotype may have a relationship to altered V-ATPase activity, but the exact mechanism remains unclear. Yeast mutants lacking all V-ATPase activity do not have greatly enlarged vacuoles, although there are defects in vacuolar morphology (Baars et al., 2007). The *vph1* mutations that prevented PI(3,5)P_2_ activation had significantly enlarged vacuoles relative to wild-type cells in the absence of salt, and less vacuolar fragmentation upon salt stress (Banerjee et al., 2019), but these phenotypes were not as pronounced as in PI(3,5)P_2_-deficient mutants. A screen for mutations able to suppress the temperature sensitivity of a *fab1Δ* mutant identified mutations in *VPH1* as well as a vacuolar cation/H^+^ antiporter, Vnx1; these suppressors also reduced vacuolar size (Wilson et al., 2018). The V-ATPase inhibitor bafilomycin A1 prevented vacuolar enlargement in a *fab1*^ts^ mutant upon a shift to high temperature, also suggesting that reducing V-ATPase activity reduced vacuolar swelling (Wilson et al., 2018). In addition, bafilomycin A1 blocks cytosolic vacuolation arising from treatment of COS7 cells with a PIKfyve inhibitor (Compton et al., 2016). This observation suggests that the relationship between the V-ATPase and vacuolation is conserved in mammalian cells. These results are not easily reconciled with PI(3,5)P_2_ activation of the V-ATPase, but in yeast, loss of PI(3,5)P_2_ and vacuolar enlargement were accompanied by elevated cellular levels of K^+^ ions (and to a less extent Na^+^ and Ca^2+^ ions) suggesting that vacuolar enlargement is a consequence of perturbed osmotic regulation (Wilson et al., 2018). In this mechanism, hyperactive uptake of ions into the vacuole, likely via H^+^-driven antiporters, drives vacuole swelling; inhibiting the V-ATPase prevents the overactive uptake by inhibiting the antiporters. There is no direct evidence that a similar mechanism explains the cytosolic vacuolation in mammalian cells lacking PI(3,5)P_2_. Notably, it has been shown that overexpression the Ca^2+^ release channel TRPML1 and its yeast homologue Yvc1 can suppress vacuolation in PI(3,5)P_2_-deficient mouse fibroblasts and yeast, possibly by altering fission and fusion events (Dong et al., 2010). Lysosomal enlargement with acute PIKfyve inhibition appears to occur via lysosomal fusion (Choy et al., 2018). Both PI(3,5)P_2_ and the V-ATPase are implicated in yeast vacuolar fission and fusion (Baars et al., 2007; Miner et al., 2019), but it is not yet clear whether they act together or in parallel.

V-ATPase activity is compromised in mutants lacking Vps34, the kinase responsible for generating PI(3)P, as well as its partner regulatory subunit Vps15 (Sambade et al., 2005). *vps34Δ* and *vps15Δ* mutants were identified as exhibiting Vma^–^ growth phenotypes in a genomic screen, and these mutants showed little uptake of quinacrine into the vacuole. Because PI(3)P is the substrate of Fab1/PIKfyve, PI(3,5)P_2_ synthesis is also lost in mutants unable to synthesize PI(3)P, but the V-ATPase defects exceeded those of the *fab1Δ* mutant in yeast. Vacuolar vesicles isolated from *vps34Δ* and *vps15Δ* mutants had only 15–17% of wild-type activity, and had reduced levels of both V_1_ and V_o_ subunits, despite the presence of normal subunit levels in whole cell lysates (Sambade et al., 2005). These data indicate an additional defect in trafficking of Vph1-containing V-ATPases in mutants lacking PI(3)P that could be explained by transport of the V-ATPase to the yeast vacuole through the prevacuolar compartment, a Vps34-dependent pathway (Bowers and Stevens, 2005). Although mammalian cells have three PI 3-kinases, the Vps34 homologue (also known as PIK3C3) plays a critical role in endosomal trafficking and autophagy and forms the predominant pool of PI(3)P that is the precursor of PI(3,5)P_2_ (Ikonomov et al., 2015). Recently, inhibition of cytosolic vacuolation in response to PIKfyve inhibition by bafilomycin was shown to involve attenuation of the elevated PI(3)P levels that generally accompany inhibition of PI(3,5)P_2_ production (Sbrissa et al., 2018). These results highlight the importance of the V-ATPase in maintaining PI(3)P levels when PIKfyve is inhibited and suggest a potential role for PI(3)P in cytosolic vacuolation beyond acting as a PI(3,5)P_2_ precursor. The direct effects of Vps34-generated PI(3)P on V-ATPase activity in mammals has yet to be examined, but there are multiple connections between PI(3)P and compartment acidification that may relate to V-ATPase function, as described below.

## Intersections of PIPs and Organelle pH in Health and Disease

Beyond direct connections to the V-ATPase, the interplay between PIP lipids and pH homeostasis occur at a very basic level. Many of the PIP lipid headgroups, including PI(3,5)P_2_ and PI(4)P have near-neutral pKa values when evaluated in a mixed lipid membrane environment (Kooijman et al., 2009). This pH sensitivity is physiologically relevant, since it was recently shown that PI(4)P exhibits cytosolic pH-sensitive interactions with protein effectors containing PH domains, including the lipid exchange protein Osh1. This pH sensitivity renders the interaction glucose-sensitive and leads to altered trafficking of proteins to the membrane in glucose-deprived cells (Shin et al., 2020). In contrast, levels of PI(3)P are sensitive to luminal pH. As the internal pH of endosomes and phagosomes decrease, Vps34 gradually dissociates from the cytosolic face of the membrane, halting PI(3)P production and rendering the lipid susceptible to phosphatases (Naufer et al., 2017). Taken together, these data highlight the intimate connection between PIP content and pH homeostasis. These connections play out in multiple settings.

### Neurodegeneration

Both PI(3,5)P_2_ and V-ATPase activity have strong associations with neurodegenerative disease. Mice with impaired PI(3,5)P_2_ synthesis exhibit severe neurodegeneration (Chow et al., 2007; Zhang et al., 2007), and mutations that reduce PI(3,5)P_2_ levels in humans are associated with Charcot-Marie-Tooth disorder 4J and amyotrophic lateral sclerosis (Chow et al., 2007; Chow et al., 2009). Fig4- and Vac14-deficient mice show early neurodegeneration that has been attributed to defects in autophagy as well as the defective trafficking leading to vacuolation (Ferguson et al., 2009). Although complete loss of V-ATPase activity is lethal in mammals (Sun-Wada et al., 2000), compromised lysosomal acidification is associated with multiple neurodegenerative diseases and aging (Colacurcio and Nixon, 2016; Song et al., 2020). One form of Batten’s disease arises from mutations in the CLN1 gene which compromise trafficking of the a1 subunit isoform and results in elevated lysosomal pH and severe neurodegeneration (Bagh et al., 2017). Mutations in LRRK2 (leucine-rich repeat kinase 2) are strongly associated with Parkinson’s disease, and LRRK2 was recently shown to interact with the a1 subunit isoform. Significantly, the pathogenic LRRK2 R1441C mutation alters interaction with a1 and increases lysosomal pH (Wallings et al., 2019). Loss of the a1 subunit has also been associated with Alzheimer’s disease (Williamson and Hiesinger, 2010). Mutations associated with altered splicing of human V-ATPase subunit gene ATP6AP2 are associated with both a specific form of Parkinson’s disease, X-linked parkinsonism with spasticity (Korvatska et al., 2013) and X-linked mental retardation with epilepsy, Hedera type (Ramser et al., 2005; Hirose et al., 2019). Conditional deletion of ATP6AP2 in mouse brain results in severe developmental defects and widespread neurodegeneration (Hirose et al., 2019), and similar defects were seen in Drosophila (Dubos et al., 2015). Autophagic flux requires V-ATPase activity both for autophagosome-lysosome fusion and autolysosome acidification/cargo degradation (Mauvezin and Neufeld, 2015). Long-lived cells like neurons are particularly sensitive to reduced lysosomal proteolysis. Oocytes are also generally long-lived cells, and interestingly, stimulation of V-ATPase activity proved to be critical for restoring oocyte proteostasis prior to fertilization in *Caenorhabditis elegans*, thus insuring a “reset” that clears any accumulated aggregates (Bohnert and Kenyon, 2017). If PI(3,5)P_2_ proves to activate V-ATPase activity in response to stress in mammalian cells as it does in yeast, this activation could be a pathway for neuroprotection.

### Phagocytosis and Immunity

As described above, proteins entering the cell via endocytosis encounter a progression of compartments with distinct luminal pH and phosphoinositide content. This progression is even more elaborate in phagocytic cells, which display both a succession of transitions in PIP lipid content and a progressively lower pH with time. Sun-Wada et al. (2009) found that V-ATPases containing the a3 subunit isoform were responsible for phagosome acidification, and were recruited to phagosomes by phagosome-lysosome fusion. The a3 isoform appeared at the phagosomal membrane by 10 min. after phagocytosis of latex beads, and remained on the membrane through later stages of phagocytosis. During these stages, the phagosomal pH decreased from 7.5 to 5.9. Both phagosome acidification and bacterial killing were lost in macrophages from a mutant mouse lacking the a3 subunit (Sun-Wada et al., 2009). Interestingly, it was recently shown that if reacquisition of PI(4)P in late phagosomes is blocked, acidification of the phagosome is compromised (Levin et al., 2017). As in a number of cases, it is not yet clear whether PI(4)P facilitates fusion with acidic lysosomes with the phagosomes at this later stage, directly affects acidification, or both. Reduced PI(3,5)P_2_ was shown to compromise phagosome-lysosome fusion and degradative capacity in RAW macrophages, but acidification of both phagosomes and lysosomes was preserved, suggesting that acidification is not dependent on this lipid (Kim et al., 2014). Dendritic cells process endocytosed proteins for antigen presentation, and increased assembly of the V-ATPase in lysosomes accompanies dendritic cell maturation and results in a lower lysosomal pH (Trombetta et al., 2003; Liberman et al., 2014). More recently, PIKfyve was shown to be critical for maturation of phagocytic compartments in dendritic cells and generation of an “MHC Class II compartment” where protein fragments are combined with MHC Class II molecules for antigen presentation. Inhibition of PIKfyve in dendritic cells impaired lysosomal acidification and antigen presentation, slowing maturation of the phagocytic compartment and reducing the activity of proteases with an acidic pH optimum (Baranov et al., 2019). In a *Dictyostelium* model of phagocytosis, loss of PI(3,5)P_2_ decreased acidification and increased survival time of *Legionella* bacteria (Buckley et al., 2019). Many pathogens, including *Legionella* and *Mycobacterium tuberculosis*, manipulate both PIP levels and organelle acidification to modify the phagocytic pathway and enhance their survival (Hilbi et al., 2011; Koliwer-Brandl et al., 2019). The cellular entry and intracellular life-cycle of SARS-Cov-2 virus was blocked both by V-ATPase and PIKfyve inhibitors in a human pseudovirus infection model, indicating an important role of ion homeostasis at the endo-lysosome in coronaviral propagation (Ou et al., 2020). Taken together, these data emphasize the importance of both PIP lipids and the V-ATPase in immunity. They indicate multiple intersections between PI(3,5)P_2_ production and the acidification of compartments required for antigen presentation and pathogen killing. Future experiments are needed to determine whether it is possible that PI(3,5)P_2_ is essential for fusion of lysosomes with the maturing phagosome in certain settings and/or directly activating the V-ATPase in others.

### Cancer

Both V-ATPases and PIPs play important signaling roles in cancer (Stransky et al., 2016; Goncalves et al., 2018), but they also exhibit critical interactions in the endomembrane system that may not be directly related to signaling. Certain cancers are addicted to autophagy, and treatment with the weak base chloroquine inhibited growth of pancreatic tumors in mice (Yang et al., 2011). The importance of V-ATPase function and Vps34-containing complexes in autophagy are described above. V-ATPase activity is upregulated in cancer, and this upregulation is associated with both drug resistance and metastasis in a number of cancers (Stransky et al., 2016). Upregulation of lysosomal V-ATPases enhances drug resistance by promoting sequestration of chemotherapeutics. In addition, three of the four mammalian a-subunit isoforms have been implicated in recruitment of their V-ATPase subpopulations to the plasma membrane. In cancer cells with oncogenic RAS activation mutations, which require metabolic adaptations, recruitment of V-ATPases containing the a3 isoform to the plasma membrane was essential for inducing nutrient uptake by macropinocytosis (Ramirez et al., 2019). In human breast cancer, upregulation of the a3 isoform is associated with increased invasion and metastasis, and V-ATPases containing a3 are recruited to the plasma membrane in cancer cells but not in normal breast epithelial cells (Cotter et al., 2016). In a mouse breast cancer model, V-ATPases containing the a4 subunit isoforms were recruited to the plasma membrane (McGuire et al., 2019). In a prostate cancer cell line, both the a1 and the a3 subunit isoforms were shown to recruit from different compartments to the plasma membrane (Smith et al., 2016). In each of these cell types, V-ATPases containing the a-subunit isoforms occupied internal compartments, where they may have contributed to cell proliferation, but were also recruited to the plasma membrane, potentially to assist with pH homeostasis and reduce extracellular pH. Interestingly, recruitment to the plasma membrane would require them to function in a very different lipid environment. Specifically, V-ATPases containing the a1 and a3 generally occupy late endosomes and lysosomes enriched in PI(3)P and PI(3,5)P_2_, and movement to the plasma membrane requires them to function in the absence of these lipids (Smith et al., 2016). It will be intriguing to see if the altered lipid environment could make these V-ATPases less responsive to certain stresses than they would be in their normal organelle environment.

### Future Directions

Recent evidence indicates that PIPs and V-ATPase both serve as critical components of organelle identity, and play central roles in pH and endomembrane homeostasis. Dissecting direct and indirect roles of PIPs in V-ATPase regulation requires both an assessment of which populations of mammalian V-ATPases show PIP-dependent localization, activity, or regulation, and generation of tools such as mutants defective in specific PIP interactions to test the importance of those functions in various cell types. Although V-ATPases are attractive therapeutic targets in diseases ranging from cancer to osteoporosis, pursuing their potential as targets has been difficult because of toxicity associated with total loss of V-ATPase activity in mammals. PIP interactions characteristic of V-ATPase subpopulations could provide a novel mechanism for inhibiting specific V-ATPase subpopulations.

## Author Contributions

SB prepared figures. SB and PK wrote and edited the review. Both authors contributed to the article and approved the submitted version.

## Conflict of Interest

The authors declare that the research was conducted in the absence of any commercial or financial relationships that could be construed as a potential conflict of interest.
